# A Sunny Face

**Published:** 1872-03

**Authors:** 


### A SUNNY FACE.

How sweet in infancy, how lovely in youth, how saintly in age! There are a few noble natures whose very presence carries sunshine with them wherever they go; a sunshine which means pity for the poor, sympathy for the suffering, help for the unfortunate, and benignity towards all. How such a face enlivens every other face it meets, and carries into every company vivacity, and joy, and gladness. But

### THE SCOWL AND FROWN, begotten in a selfish heart, and manifesting itself in daily, almost hourly fretfulness, complaining, fault-finding, angry criticisms, spiteful comments on the motives and actions of others, how they thin the cheek, shrivel the face, sour and sadden the countenance! No joy in the heart, no nobility in the soul, no generosity in the nature; the whole character as cold as the iceberg, as hard as an Alpine rock, as arid as the wastes of Sahara! Reader, which of these countenances are you cultivating? If you find yourself losing all your confidence in human nature, you are nearing an old age of vinegar, of wormwood, and of gall; and not a mourner will follow your solitary bier, not one tear-drop shall ever fall on your forgotten grave.

